# *Turnip Mosaic Virus* Transcriptional Slippage Dynamics and Distribution in RNA Subpopulations

**DOI:** 10.1094/MPMI-03-22-0060-R

**Published:** 2022-09-14

**Authors:** Kairi Kärblane, Andrew E. Firth, Allan Olspert

**Affiliations:** 1 Department of Chemistry and Biotechnology, Faculty of Science, Tallinn University of Technology, Tallinn, Akadeemia tee 15, 12618, Estonia; 2 Division of Virology, Department of Pathology, University of Cambridge, Addenbrooke’s Hospital, Hills Road, Cambridge CB2 0QQ, U.K.

**Keywords:** NMD, PIPO, PISPO, potyvirus, transcriptional slippage

## Abstract

Potyviruses comprise the largest and most important group of plant positive-strand RNA viruses. The potyviral cell-to-cell movement protein P3N-PIPO is expressed via transcriptional slippage at a conserved GAAAAAA sequence, leading to insertion of an extra ‘A’ in a proportion of viral transcripts. Transcriptional slippage is determined by the potyviral replicase, the conserved slippery site, and its flanking nucleotides. Here, we investigate the dynamics of transcriptional slippage at different slip-site sequences, infection stages, and environmental conditions. We detect a modest increase in the level of transcripts with insertion towards later timepoints. In addition, we investigate the fate of transcripts with insertion by separately looking at different RNA subpopulations: (+)RNA, (−)RNA, translated RNA, and virion RNA. We find differences in insertional slippage between (+)RNA and (−)RNA but not other subpopulations. Our results suggest that there can be selection against the use of (−)RNAs with insertions as templates for transcription or replication and demonstrate that insertional slippage can occur at high frequency also during (−)RNA synthesis. Since transcripts with insertions are potential targets for degradation, we investigate the connection to nonsense-mediated decay (NMD). We find that these transcripts are targeted to NMD, but we only observe an impact on the level of transcripts with insertion when the insertional slippage rate is high. Together, these results further our understanding of the mechanism and elucidate the dynamics of potyviral transcriptional slippage.

Viruses have evolved many unusual gene-expression strategies in order to maximize the coding output from compact genomes. Many of these mechanisms impact translation and are sometimes referred to as ribosome gymnastics (Atkins et al. 1990). The best-characterized examples are programmed ribosomal frameshifting, programmed stop-codon readthrough, internal ribosome entry, and ribosomal skipping (Firth and Brierley 2012). RNA viruses have also evolved non-canonical mechanisms of transcription for the expression of certain proteins. Transcriptional slippage, sometimes also labeled with confusing terms such as ‘transcriptional editing’ or even ‘RNA editing’, is a process in which one or both the RNA polymerase and nascent strand slips and loses nucleotide register on the template strand in a controlled manner, which results in the addition or skipping of nucleotides in the newly synthesized RNA. This enables access to alternative reading frames with canonical translation of these products. The best-known examples come from the negative-sense single-stranded RNA virus families *Paramyxoviridae* and *Filoviridae*, in which prominent members, such as measles virus and Ebola virus, respectively, utilize this mechanism (Cattaneo et al. 1989; Sanchez et al. 1996). Positive-sense RNA viruses also use this mechanism, with members of family *Potyviridae* currently the only known examples (Hagiwara-Komoda et al. 2016; Olspert et al. 2015; Rodamilans et al. 2015; Untiveros et al. 2016). In addition to providing access to additional reading frames, both translational and transcriptional recoding mechanisms enable viruses to optimize the ratio between alternative protein products via the efficiency of the noncanonical expression mechanism.

Potyviruses are plant viruses with flexuous filamentous virions and single-stranded positive-sense RNA genomes approximately 10 kb in length (Revers and García 2015). The 5′ end of a potyviral genome contains a covalently linked protein (VPg) and the 3′ end has a poly(A) tail. Potyviruses do not use subgenomic RNAs and most viral proteins are translated from the genome as a single long polyprotein, which is cleaved by viral proteases to produce the mature proteins. All potyviruses contain an overlapping open reading frame (ORF) in the middle of the P3-encoding region of the polyprotein ORF in the −1/+2 reading frame, which is named *pipo* (Chung et al. 2008). In addition, the *Sweet potato feathery mottle virus* subgroup of potyviruses also have an overlapping ORF in the P1-encoding region in the −1/+2 frame, which is named pispo (Clark et al. 2012; Li et al. 2012). These overlapping ORFs are expressed as C-terminal fusions with their corresponding polyprotein products, P3N-PIPO and P1N-PISPO, which have virus cell-to-cell movement and RNA silencing suppression activities, respectively (Mingot et al. 2016; Untiveros et al. 2016; Vijayapalani et al. 2012; Wen and Hajimorad 2010).

Despite the economic importance of potyviruses, our knowledge of the particular mechanism of transcriptional slippage is limited. Both *pipo* and *pispo* overlapping ORFs start at highly conserved GAAAAAA sequences that signal the viral polymerase to add an extra ‘A’ nucleotide in a proportion of transcripts. Insertion efficiencies at the *pipo* slip site have been reported to be around 1 to 2%, while at the *pispo* slip site, frequencies in the range 5 to 12% have been observed (Olspert et al. 2015; Rodamilans et al. 2015; Untiveros et al. 2016). We have previously shown that the nucleotide sequence immediately adjacent to the GAAAAAA modulates slippage efficiency and we have not previously found evidence supporting more complex regulation of slippage efficiency (Olspert et al. 2016). We have demonstrated that increased GC content up- or downstream, or both, of the homopolymeric hexamer (HH) correlate with higher insertion levels, the conserved G is not strictly required for slippage, and a homopolymeric run of six nucleotides is optimal for mostly single-nucleotide insertions (Olspert et al. 2016). In addition, we have identified a process termed ‘to-fro’ slippage, which occurs only with certain nucleotides flanking the HH. During to-fro slippage, the −1 or +7 nucleotide position relative to the start of HH (Fig. 1A) suffers a templated substitution during negative RNA [(−)RNA] or positive RNA [(+)RNA] synthesis, respectively (Olspert et al. 2016). This process is mechanistically related to insertional slippage and seems to compete with insertional slippage. In both cases the original template register is lost with a minus direction slip, but with to-fro slippage, the original register is restored after insertion of a single templated nucleotide (Olspert et al. 2016). To-fro slippage depends on the template nucleotide at the −1 or +7 nucleotide position and the adjacent nucleotide in the HH both being purines or both being pyrimidines so that, following realignment, the mismatch between the template and nascent RNAs will be purine:pyrimidine or pyrimidine: purine rather than purine:purine or pyrimidine:pyrimidine.

Since there is selection against six ‘A’s or six ‘U’s in potyviral and picornaviral genomes in general, the possibility of wider use of transcriptional slippage in positive-strand RNA viruses has been proposed (Hagiwara-Komoda et al. 2016; Stewart et al. 2019). Evidence from picornaviruses show insertional slippage can occur at potyviral slip sites, but the insertion levels and other characteristics differ considerably from potyviral data, suggesting that the potyviral RNA polymerase (NIb protein) has evolved to functionally slip at these nucleotide motifs (Stewart et al. 2019). In recent years, in addition to RNA silencing suppression, potyvirus-host interactions influencing general RNA degradation pathways have been characterized (Li and Wang 2018). VPg and HC-Pro interact with DCP2 and XRN4, respectively, to compromise their antiviral function in RNA decay pathways (Li and Wang 2018). Insertional slippage produces transcripts with long, about 6-kb 3′ untranslated regions (UTRs) (about 9 kb for an artificially inserted slip site, 2ndGA6, that was utilized in this study), and it is known that long 3′ UTRs operate as *cis*-acting elements to trigger RNA nonsense-mediated decay (NMD) in plants (Kertész et al. 2006). Some plant and mammalian viral RNAs are known targets of NMD (Balistreri et al. 2014; Garcia et al. 2014). Thus, NMD might be a negative regulator of the levels of potyviral transcripts with insertions (TWI).

In the current study, we investigate slippage dynamics and the fate of TWI within different turnip mosaic virus (TuMV) RNA subpopulations. Using infection time series at different plant-growth temperatures, we establish TuMV transcriptional slippage as a dynamic process, dependent on infection stage and environmental conditions. We also show that native genomes and TWI are not treated differently when it comes to translation and packaging into virions. Furthermore, our results indicate that slippage can also occur at high frequency during negative-strand synthesis and during synthesis of homopolymeric ‘U’ stretches. However, our data suggest that (−)RNAs with insertion might be selected against in subsequent rounds of transcription and replication. We also find that, although NMD is probably involved in regulating the levels of TWI, the impact of NMD on TWI levels or on viral infection is only noticeable when high amounts of TWI are produced.

## Results

### Characterization of slip-site mutants

**Fig. 1:**
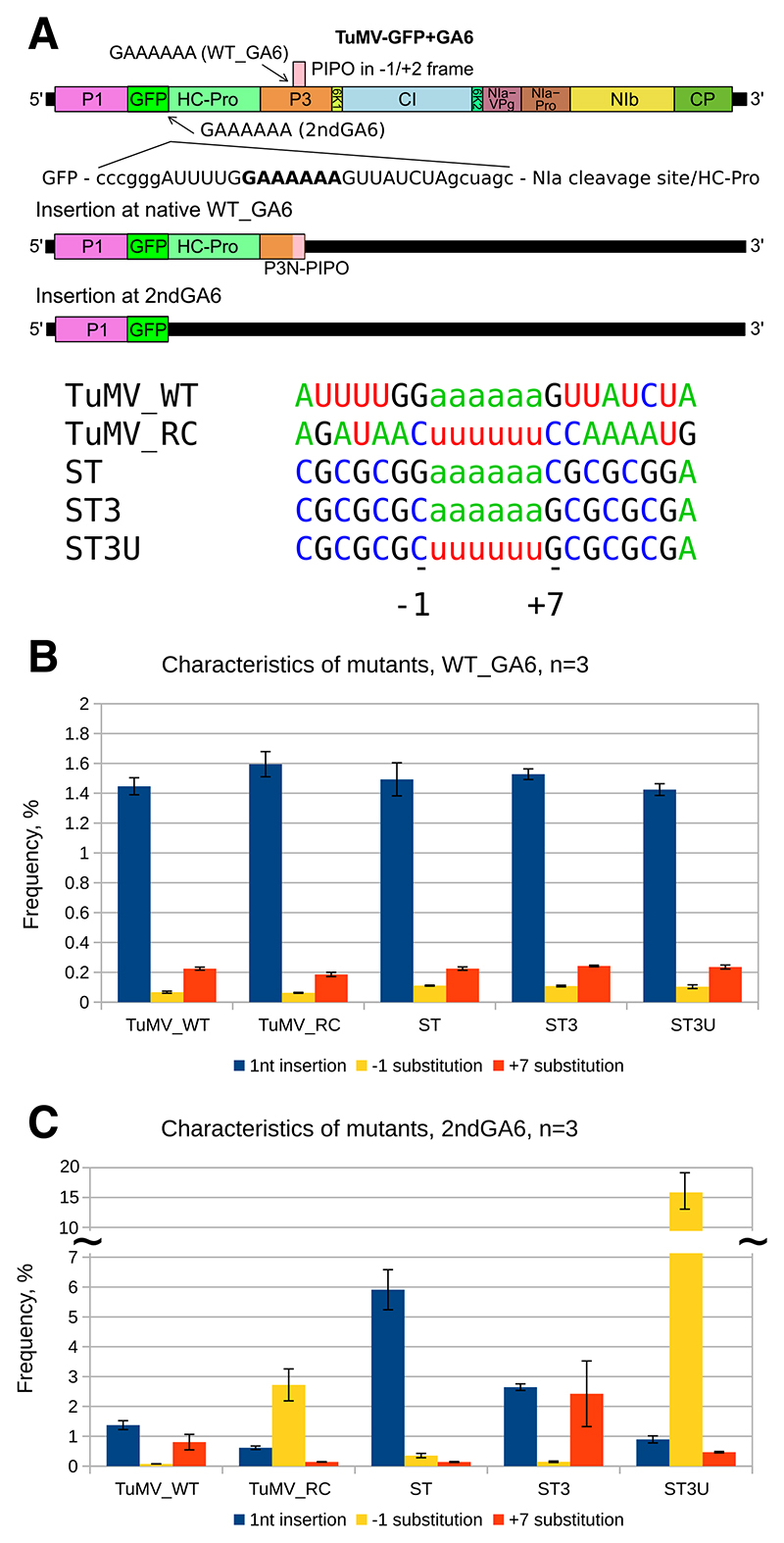
Overview of slip-site mutants with detected insertion and substitution frequencies at key positions. **A**, Schematic of the turnip mosaic virus (TuMV) genome with green fluorescent protein (GFP) and the additional inserted 2ndGA6 slip site. Colored boxes indicate the polyprotein cleavage products. The GFP sequence between P1 and HC-Pro is followed by a small insert containing an additional GAAAAAA slip-site sequence with flanking nucleotides (2ndGA6) and restriction nuclease *Sma*I and *Nhe*I recognition sites (lower case) enabling manipulation. The 2ndGA6 insert is shown below the genome diagram (TuMV wild-type (WT) sequence shown). The position of the conserved GAAAAAA sequence at the 5′ end of the pipo open reading frame (WT_GA6) is indicated with an arrow. The coding capacity of transcripts with a single nucleotide insertion at either slip site is shown below, followed by TuMV mutant names and corresponding 2ndGA6 sequences. Positions −1 and +7 with respect to the slippery hexamer are indicated at the bottom. **B and C**, *Nicotiana benthamiana* plants were inoculated with TuMV constructs and systemically infected leaves were harvested at 10 days postinoculation. Total RNA was extracted and was subjected to reverse transcription PCR and amplicon high-throughput sequencing; three biological samples were used (*n* = 3). B and C correspond to the native *pipo* slip site (WT_GA6) and the artificial slip site (2ndGA6), respectively. Single nucleotide insertions in the slippery homohexamer, producing a homoheptamer, are in blue; substitutions at the −1 and +7 positions are in yellow or orange, respectively. Error bars indicate standard deviation. A discontinuous *y* axis is indicated with tildes (~).

In order to further our understanding of the mechanism of transcriptional slippage and the biogenesis of TWI, we used the previously described TuMV-GA6, the green fluorescent protein (GFP)-expressing infectious clone of the potyvirus TuMV with an additional slip site inserted at the 3′ end of the *gfp* coding sequence (Olspert et al. 2016). This enables the simultaneous study of slippage at the native *pipo* slip site (WT_GA6) and at the introduced site (2ndGA6) independently within a single infection. The sequence of the 2ndGA6 site is free of overlapping coding restrictions so that, in principle, any kind of slip sequence can be used as long as in-frame stop codons are not introduced, whereas the WT_GA6 site is identical for all tested viruses. Five TuMV mutants with different 2ndGA6 slip sites were chosen for the current study (Fig. 1A). Virus clones named after the 2ndGA6 sequence are as follows: TuMV_WT, the sequence corresponding to the TuMV native *pipo* slip site; TuMV_RC, reverse complement of the TuMV native *pipo* slip site; ST, an artificial slip site with a high single-nucleotide insertion level; ST3, an artificial C/G-rich site that is identical in positive and negative sense except for the HH itself; and ST3U, the reverse complement of ST3 (i.e., ST3 with a U6 instead of A6 hexamer in the sense orientation). TuMV_WT, TuMV_RC, and ST have been previously characterized (Olspert et al. 2016). ST3 and ST3U were generated for the current study in order to analyze slippage with identical upstream and downstream sequences in both (+)RNA and (−)RNA.

Slippage was assessed via high-throughput sequencing of targeted amplicons and quantification of insertions and substitutions relative to unmodified sequences (Olspert et al. 2016). As expected, insertional and to-fro slippage levels were similar between the native slip sites of all tested viruses (Fig. 1B). At the 2ndGA6 site (Fig. 1C), TuMV_WT displayed similar insertional slippage to the native site but an elevated substitution frequency at the +7 position, TuMV_RC had a reduced insertional slippage level and an elevated −1 substitution level, and ST had a high insertional slippage level. These observed slippage characteristics are similar to previous observations (Olspert et al. 2016). Mutant ST3 displayed an insertion level of 2.7% (± 0.1%) and an elevated substitution level at position +7, whereas, compared with ST3, mutant ST3U had a reduced insertion level of 0.9% (± 0.1%) and a considerably elevated substitution level at the −1 position, reaching 16.1% (± 3.1%). The slippage characteristics of ST3 and ST3U imitate those of the TuMV_WT and TuMV_RC constructs, respectively, but at an amplified scale, resulting in better detection and reflecting the previously observed trend for increased slippage with increased GC content up- or downstream, or both, of the slip site (Olspert et al. 2016). Although the substitution frequencies are different and the sub-stitution characteristics depend on specific sequences, in most cases, positions −1 and +7 were more frequently substituted by the nucleotide corresponding to the proximal HH to A or U (Supplementary Table S1), similar to what was noted previously (Olspert et al. 2016).

### Environmental conditions and the infection stage influence the insertional slippage level at WT_GA6

**Fig. 2:**
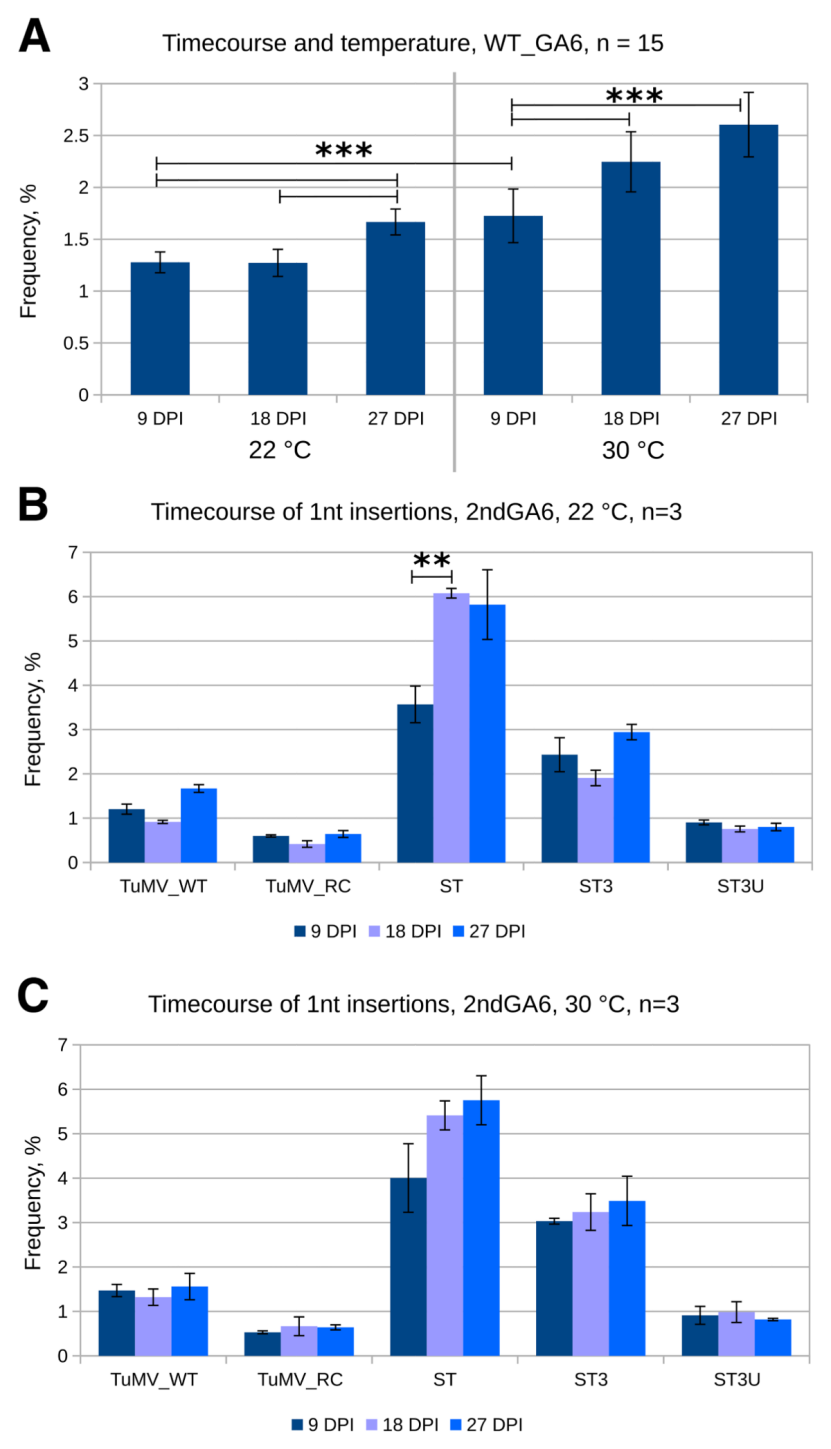
Transcriptional slippage during infection at different temperatures. *Nicotiana benthamiana* plants were inoculated with turnip mosaic virus (TuMV) constructs and were grown at 22 or 30°C. Systemically infected top leaves and flower buds were harvested 9, 18, and 27 days postinoculation (DPI). Total RNA was extracted and subjected to reverse transcription PCR and amplicon high-throughput sequencing; three biological samples were used for each treatment (*n* = 3). **A**, Insertional slippage at the native *pipo* slip site WT_GA6. **B and C**, Insertional slippage at the artificial slip site 2ndGA6, at different timepoints, for plants grown at 22°C (B) and 30°C (C). Error bars indicate standard deviation. Statistically significant differences (analysis of variance, Tukey highly significant difference: *P* < 0.01 [**], *P* < 0.001 [***]) between single nucleotide insertion levels are indicated when relevant.

Slippage dynamics during infection and slippage in stress conditions were investigated in a time series of plants grown at normal (22°C) or heat stress (30°C) temperatures. The systemically infected top leaves and flower buds were harvested for analysis at 9, 18, and 27 days postinoculation (DPI) (Fig. 2). At 22°C, the insertion level at WT_GA6 was similar in 9- and 18-DPI samples, whereas, in 27-DPI samples, the insertion levels were significantly higher although the change was small (from 1.3% ± 0.1% at 9 DPI to 1.7% ± 0.1% at 27 DPI) (Fig. 2A). At 30°C, a significantly elevated insertion level, 1.7% ± 0.3%, was already observed at 9 DPI (elevated compared with 9 DPI at 22°C) and it continued to increase at later timepoints. However, differences in infection progression and plant response under different conditions need to be taken into account when interpreting and comparing these data. Plants grown at 22°C are overwhelmed by infection by 9 DPI when the virus has colonized and is replicating in the top part of the plant (growth is almost halted), whereas plants grown at 30°C are considerably more tolerant to the infection and continue to grow after 9 DPI (Supplementary Fig. S1). Therefore, all timepoints from 22°C represent practically the same tissue and are comparable at different infection stages but, between temperatures, only the 9-DPI sample from 30°C is comparable to the matched timepoint at 22°C. Later samples obtained at 30°C show the slippage at the continuously growing shoot top, which corresponds to later infection stages but also in new tissue. Nonetheless, it is clear from the 18- and 27-DPI samples from 30°C that significantly different insertion frequencies can be detected, depending on both infection stage and altered host homeostasis at elevated temperature, in comparison to 22°C.

At the 2ndGA6 site, the insertional slippage level depended on the slip-site sequence but dependence on the infection stage or growth conditions was not obvious (Fig. 2B and C). Apart from the ST 9-DPI values, the insertion levels were characteristic of a particular slip sequence and fluctuated modestly between the samples of different infection stage and growth temperature. At both temperatures, the ST 9-DPI values were lower than expected, but later timepoint values of ST settled near the previously observed level. Although statistically significant, the changes at WT_GA6 were also small. Therefore, due to the low number of samples (*n* = 3 vs. *n* = 15), it is not possible to completely rule out an effect of either temperature, infection stage, or both on slippage also at the 2ndGA6 site. However, it is clear that the slippage sequence itself has the greatest effect.

Substitution profiles at the −1 and +7 positions at both slipsite locations were similar to previous data (Fig. 1B and C) across timepoints and both temperatures (data not shown). We also noticed considerable differences in relative viral RNA levels in this particular experiment, reaching close to 10-fold between different viruses at 22°C and up to 100-fold between the same virus at different temperatures (Supplementary Fig. S2). On average, there was 10-to 100-fold less virus at 30°C than at 22°C, which is in accordance with higher tolerance seen at 30°C. However, these variations in viral RNA levels between viruses (Supplementary Fig. S2) seem to have no effect on insertional slippage (Fig. 2A).

### Evidence of unequal distribution of insertions between (+)RNA and (−)RNA populations

During positive-strand RNA virus replication, a (**−**)RNA is produced from (+)RNA within a viral replication complex (VRC), after which more (+)RNAs are produced from the (−)RNA template. Subsequently, the newly synthesized (+)RNA released from a VRC may be used in at least the following ways: as template for (−)RNA synthesis in a new VRC, translated by ribosomes, packaged into virions, or degraded by either host defense pathways or general RNA degradation pathways (Mäkinen and Hafren 2014). To investigate whether viral or cellular pathways treat TWI differently from native genomes, plants were infected with the previously described viruses and different RNA subpopulations were analyzed. Total cellular RNA, virions, and polysomes were purified from the same systemically infected plant tissue, after which RNA was extracted from the latter two preparations. Strand-specific libraries for the detection of (+)RNA and (−)RNA were produced from total RNA with validated strand specificity (discussed below), and libraries from virion and polysome-associated RNA were produced identically to the (+)RNA libraries.

**Fig. 3:**
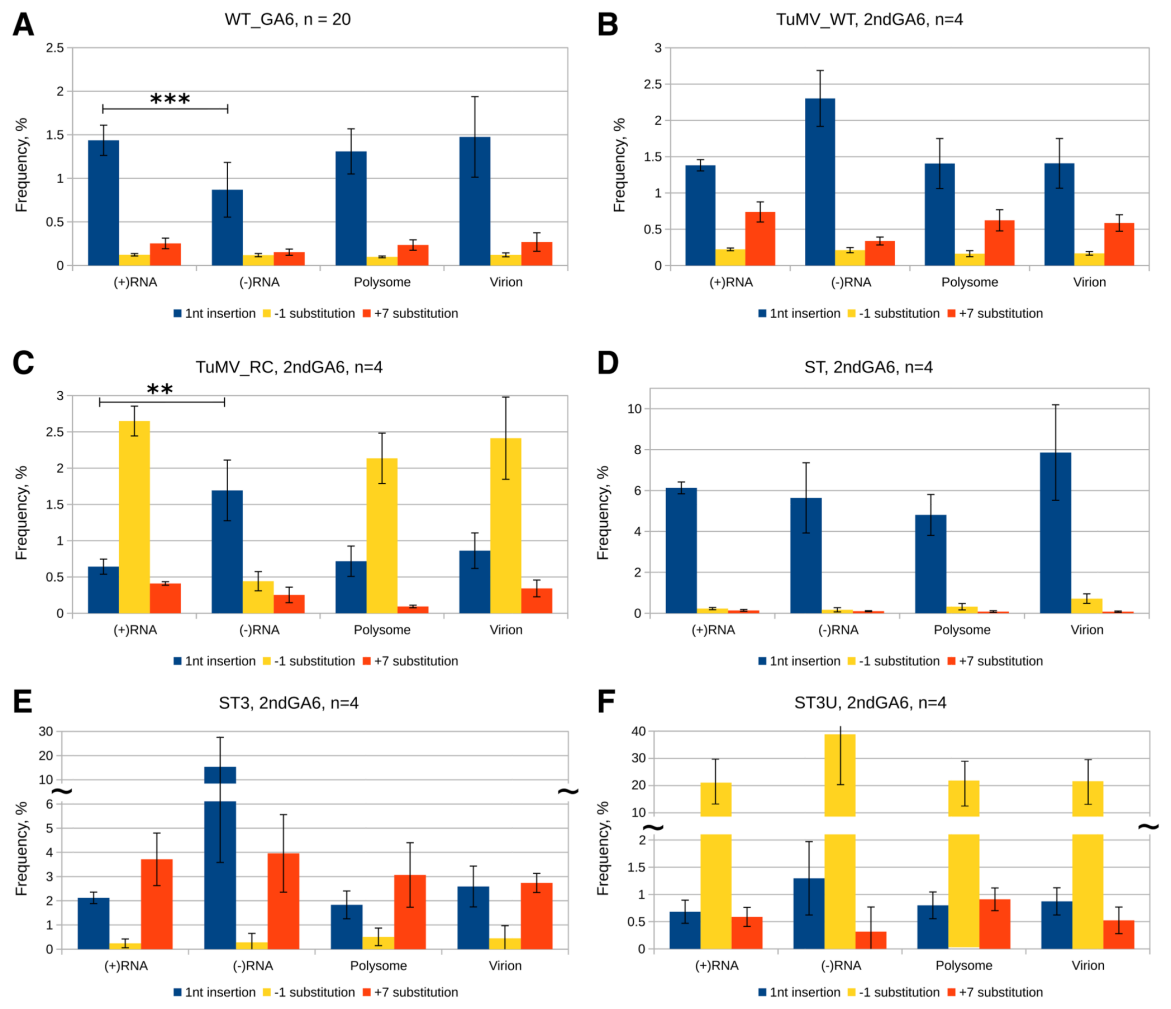
Distribution of transcriptional slippage products within RNA subpopulations. *Nicotiana benthamiana* plants were inoculated with turnip mosaic virus (TuMV) constructs and systemically infected top leaves were harvested 10 days postinoculation. Total RNA, polysomes, and virions were extracted from the same sample and were subjected to reverse transcription PCR and amplicon high-throughput sequencing; four biological samples were used (*n* = 4). **A**, Insertional and *to-fro* slippage at the native *pipo* slip site WT_GA6. **B to F**, Transcriptional slippage at the 2ndGA6 slip site of different mutants. Single nucleotide insertions in the slippery homohexamer are in blue, substitutions at −1 and +7 positions are in yellow or orange, respectively. Error bars indicate standard deviation. Statistically significant differences (analysis of variance, Tukey highly significant difference *P* < 0.01 [**], *P* < 0.001 [***]) between single nucleotide insertion levels are indicated when relevant. Discontinuous *y* axes are indicated with tildes (~).

At the WT_GA6 site, there was little difference between the observed insertion levels for (+)RNA, polysome, and virion samples but significantly less TWI were seen in the (−)RNA pool compared with others (Fig. 3A). The (+)RNA, polysome, and virion samples are also indistinguishable from each other at the 2ndGA6 site (Fig. 3B and F). However, when comparing the differences between the insertion levels of (+)RNA and (−)RNA at the 2ndGA6 site, there is a clear trend of (−)RNA having a higher fraction of TWI, except for mutant ST. With ST3, the amount of TWI in the (−)RNA pool is consistently high and several fold higher than for (+)RNA, but, unfortunately, variability is also high, with the individual samples having insertion frequencies of 7, 8, 10, and 36% (Fig. 3E), which are not correlated with (low) read counts. The data from both sites suggest that there are no significant mechanisms for excluding or enhancing the inclusion of TWI for either packaging into virions or association with ribosomes; TWI are translated or packaged, or both, as efficiently as native genomes.

**Fig. 4:**
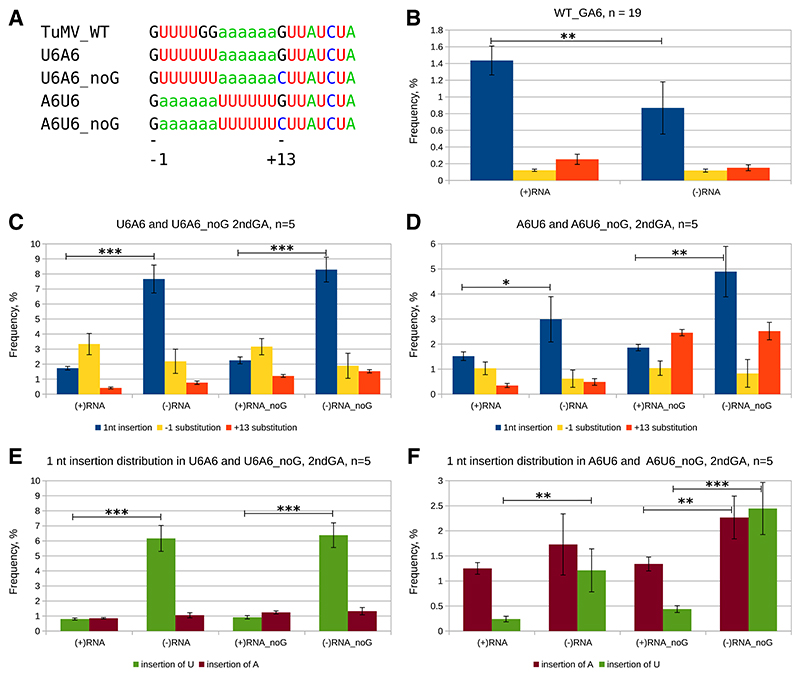
Distribution of transcriptional slippage products between (+)RNA and (−)RNA populations for double-slip mutants. *Nicotiana benthamiana* plants were inoculated with turnip mosaic virus (TuMV) constructs and systemically infected top leaves were harvested 10 days postinoculation. Total RNA was extracted and subjected to reverse transcription PCR and amplicon high-throughput sequencing; five biological samples were used (*n* = 5). **A**, Overview of slip-site sequences. Positions −1 and +13 with respect to the double-hexamer are shown at the bottom. **B**, Transcriptional slippage at the native *pipo* slip site WT_GA6. **C and D**, Transcriptional slippage at the 2ndGA6 slip site of U6A6 and A6U6 and their _noG variants. Single nucleotide insertions in either slippery homohexamer are in blue, and substitutions at the −1 and +13 positions are in yellow and orange, respectively. **E and F**, Distribution of 1-nt insertions within the two homohexamers individually (insertion of A in A6 or U in U6) at the 2ndGA6 slip site of U6A6 and A6U6, and their _noG variants. Insertion of U in the run of ‘U’s—green; insertion of A in the run of ‘A’s—brown. Error bars indicate standard deviation. Statistically significant differences (*t* test, *P* < 0.05 [*], *P* < 0.01 [**], *P* < 0.001 [***]) between single nucleotide insertion levels are indicated.

In order to better understand the slippage mechanism and confirm the detected differences in insertions between (+)RNA and (**−**)RNA, we generated four more 2ndGA6 variants based on the TuMV native *pipo* slip-site sequence (Fig. 4A). We substituted ‘GG’ before the slippery hexamer to ‘UU’, thus generating a double-slip site, U6A6. We also swapped the two hexamers, generating A6U6. In addition, the ‘G’ after the last hexamer, position 13 (equivalent of 7 in the single-slip site) was swapped to ‘C’ in the two double-slip variants to give rise to the derivatives named noG. Comparing the insertion levels between (**−**)RNA and (+)RNA again demonstrated less TWI in (−)RNA compared with (+)RNA at the WT_GA6 site but more in (−)RNA, as compared with (+)RNA at the 2ndGA6 site (Fig. 4B and D). The double-slip nature of these mutants enabled us to compare the two tandem slippery homopolymer sequence combinations within the same context and the same RNA molecule. To avoid confusion, we refer to the motifs and detected insertions discussed below in sense orientation (i.e., the text “insertion of a ‘U’ in the (−)RNA of mutant U6A6” equates to a U7A6 sequence in the sense orientation, not insertion of a ‘U’ during negative strand synthesis). The overall single-nucleotide insertion level within the two hexamers, insertion of ‘U’ into U6 or ‘A’ into A6, was modest and similar in the (+)RNA of all tested sequences, from 1.5% ± 0.2% to 2.3% ± 0.2% (Fig. 4C and D). However, in the (−)RNA, U6A6 had a higher insertion level than A6U6, at 7.6% ± 0.9% and 3.0% ± 0.9% respectively, with the noG variants behaving similarly (Fig. 4C and D). Looking at ‘A’ or ‘U’ insertions separately revealed that with U6A6 and U6A6_noG insertions in either hexamer were roughly equal in (+)RNA, but significantly more insertions of ‘U’s were seen in the (−)RNA (Fig. 4E). Clearly, only the increased insertion of ‘U’s in the latter was responsible for the overall increase in single nucleotide insertions in (**−**)RNA in comparison to (+)RNA. With A6U6 and A6U6_noG again the insertion of ‘U’s showed the most significant increase in (−)RNA compared with (+)RNA (Fig. 4F).

### Transcripts with insertion are associated with NMD

**Fig. 5:**
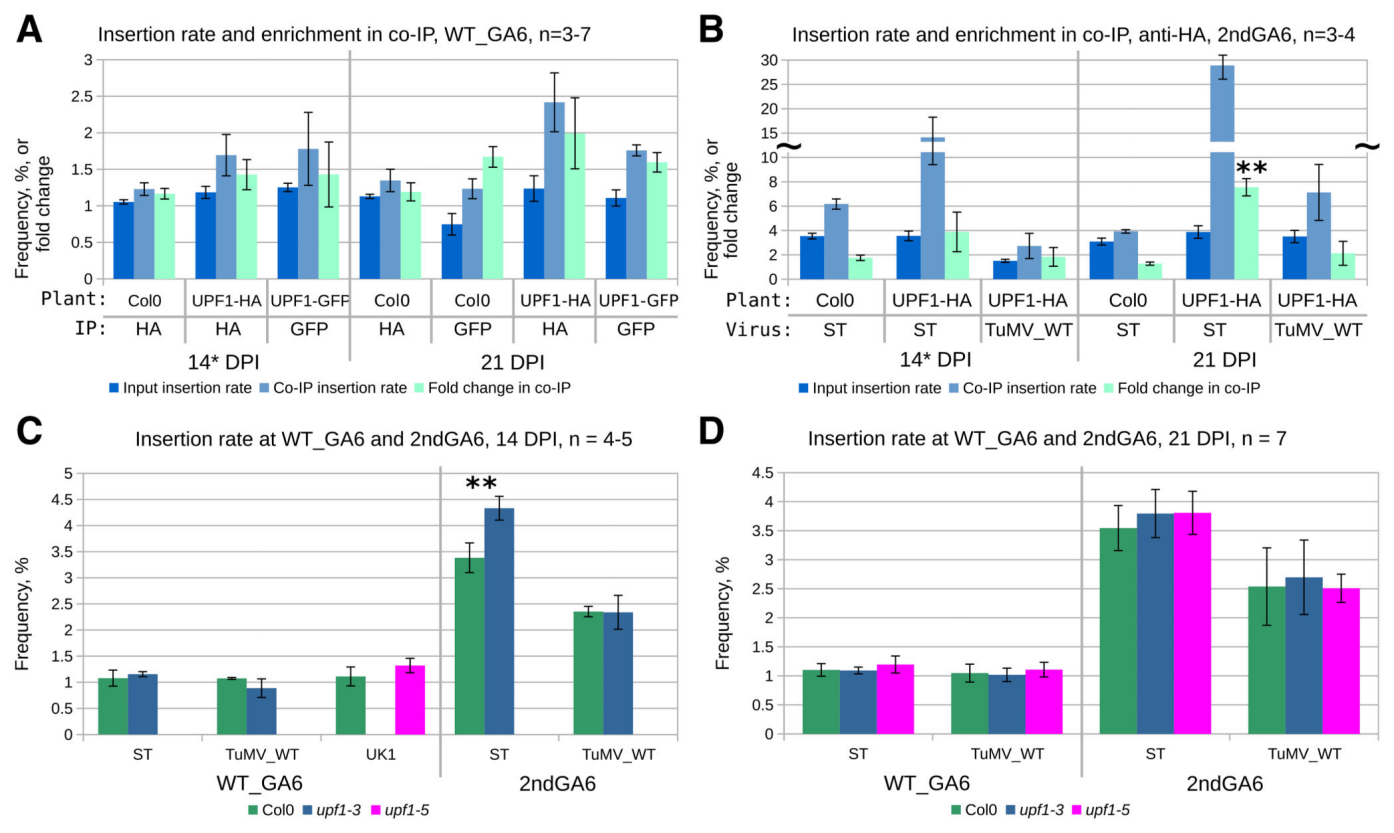
Analysis of UPF1-associated RNAs and transcriptional slippage in UPF1 knockdown lines. *Arabidopsis thaliana* plants were inoculated with turnip mosaic virus (TuMV) and systemically infected top leaves were harvested 14 or 21 days postinoculation (DPI); the 21-DPI samples also contained systemically infected stems and flowers. **A and B**, Insertional slippage levels at the WT_GA6 or 2ndGA6 slip sites, for transcripts detected in association with UPF1. Plants expressing hemagglutinin (HA)- or green fluorescent protein–tagged UPF1 were infected with TuMV; Col-0 plants were used as an untagged negative control. Homogenized infected material was used for RNA extraction (input) and co-immunoprecipitation (Co-IP) with corresponding antibody, followed by reverse transcription PCR and amplicon high-throughput sequencing. The insertion frequency (%, input or Co-IP) and fold change in Co-IP (percent Co-IP divided by percent input) are both indicated on the *y* axis; 14* denotes that two of the seven UPF1-HA (WT_GA6) and two of the four TuMV_WT (2ndGA6) were sampled at 15 DPI. **C and D**, Insertional slippage at the WT_GA6 and 2ndGA6 slip sites in the UPF1 T-DNA knockdown lines *upf1-3* and *upf1-5*. Col-0 plants were used as reference. UK1 corresponds to TuMV isolate UK1 without inserted *gfp* or the 2ndGA6 site. Col-0 is shown in green, *upf1-3* in blue, and *upf1-5* in pink. Error bars indicate standard deviation. Statistically significant differences (*t* test *P* < 0.01 [**]) between single nucleotide insertion levels are indicated. A discontinuous *y* axis is indicated with tildes (~). The number of biological samples (*n*) used is shown above each panel.

We next investigated whether TuMV TWI are targeted by NMD more frequently than native genomes. UPF1 (UP-Frameshift-1) is a universally conserved eukaryotic ATP-driven RNA helicase involved in NMD and removal of mRNAs. We infected transgenic *Arabidopsis thaliana* plants expressing UPF1 tagged with hemagglutinin (HA) or GFP (UPF1-HA, genetic background T-DNA insertion line *upf1-3*; UPF1-GFP, genetic background T-DNA insertion line *upf1-5*; both T-DNA lines are in ecotype Columbia 0 [Col-0] [Chicois et al. 2018]) with TuMV_WT, ST, or UK1 (UK1 TuMV lacks both the *gfp* and 2ndGA6 inserts, allowing utilization of UPF1-GFP). UPF1-associated RNAs were co-immunoprecipitated and analyzed for insertions (Fig. 5A and B). Col-0 expressing native untagged UPF1 was used as a negative control for co-immunoprecipitation (Co-IP). At the WT_GA6 site, we could see enrichment for TWI in Co-IP in comparison to the input for both HA and GFP, but the difference was modest and similar to the enrichment seen with Col-0 (Fig. 5A). At the 2ndGA6 site, ST—which has a higher base insertion level than WT—displayed higher enrichment in Co-IP, with later timepoint fold changes even significantly different from Col-0 (Fig. 5B). This suggests that, if there are a lot of TWI, as with ST, these are targeted more frequently by UPF1 than native genomes and, presumably, are marked for degradation.

Infection was also tested in two UPF1 T-DNA knockdown *Arabidopsis thaliana* lines, i.e., heterozygous *upf1-3* (genetic background of UPF1-HA expressing line) and homozygous *upf1-5* (genetic background of UPF1-GFP expressing line). No differences in the insertion levels between the control (Col-0) and knockdown lines were observed at either slip site with modest insertion levels (Fig. 5C and D). However, again with virus ST, which has a high baseline insertion level at 2ndGA6, slightly but significantly more insertions were detected at that site in the *upf1-3* knockdown line in the 14-DPI samples only. This again suggests the potential of UPF1 and NMD in modulating the detected insertion level when the base level of TWI is high and perhaps only at certain infection stages, as no differences were detected in the 21-DPI samples (Fig. 5D). However, despite the increase in insertion level at 14 DPI, there is no or only a very modest impact on the total level of viral RNA in UPF1 knock-down plants at any infection stage (Supplementary Fig. S3).

## Discussion

### Current understanding of the slippage mechanism

The characteristics of the utilized mutants (Fig. 1) again confirm the previously proposed slippage model, in which insertional slippage occurs mainly during the synthesis of an A hexamer (natively during positive-strand RNA synthesis) and preference of to-fro slippage over insertional slippage when the A hexamer is followed by suitable nucleotides in the opposite strand. The data also further corroborate that the conserved ‘G’ before the A hexamer is dispensable for insertional slippage and that to-fro slippage occurs when the A hexamer is followed by ‘G’, resulting in substitutions at −1 or +7 when theA hexamer is in the negative or positive strand, respectively. The ‘C’ to ‘G’ change between the ST and ST3 mutants at the +7 position appears to switch half of the insertional slippage of ST into +7 substitution to-fro slippage in ST3 (Fig. 1), consistent with insertional slippage being in competition with to-fro slippage and the latter being promoted when the post-realignment mispairing between the template and nascent RNAs retains pyrimidine:purine juxtaposition.

Slippage is determined by three key components, namely, the HH, the proximal nucleotides, and the replication complex, which behaves differently during (+)RNA or (−)RNA synthesis (Olspert et al. 2016). We detected less TWI in (−)RNA than in (+)RNA at the WT_GA6 site and, in most cases, the opposite at the 2ndGA6 site (Figs. 3 and 4). Despite the differences between the insertion levels of (+)RNA and (−)RNA being in opposite direction at the two slip sites, ‘+’ > ‘−’ at WT_GA6 and ‘+’ < ‘−’ at 2ndGA6, potentially a single explanation is plausible. The replication of (+)RNA TWI are inhibited by a known mechanism (details below) (Mahajan et al. 1996). We hypothesize that an additional mechanism may exist that in some way inhibits further transcription and replication of (−)RNA with an insertion. Due to rigorous efforts to ensure strand specificity, we can also rule out the possibility of cross-contamination between (+)RNA and (−)RNA libraries. At the 2ndGA6 site, we consistently detected more insertions in (−)RNA than in (+)RNA for different viruses (Figs. 3B, C, and E and 4C and F), which seems to support the notion of *de novo* origin of TWI during (−)RNA synthesis, as it is difficult to imagine how (+)RNA TWI might be preferentially utilized for replication. On the contrary, there is evidence of less-efficient replication of transcripts that are not translated all the way to the 3′ proximal coat protein–encoding region of the polyprotein ORF, which contains RNA secondary structures that are altered by translation (Mahajan et al. 1996). Our TWI are translated until the end of the PIPO ORF at most, which should reduce their potential for being replicated. Of course, the mechanism of (−)TWI production (proportion produced *de novo* vs. proportion replicated from a (+)TWI template) might vary with regards to one or both the slip-site sequence and slip-site location. The disbalance of ‘U’ or ‘A’ insertions between (−)RNA and (+)RNA for the U6A6 and A6U6 mutants also supports the notion of *de novo* origin of insertions in (−)RNA, at least for the 2ndGA6 site of these viruses (Fig. 4C and F). On the other hand, at the WT_GA6 site, it appears that insertions occur preferentially during (+)RNA synthesis (Figs. 3A and 4B).

Previous observations based on only (+)RNA analysis suggested that insertional slippage was favored during synthesis of ‘A’s rather than ‘U’s, implying that the viral polymerase has evolved for insertional slippage at GAAAAAA during (+)RNA synthesis (Olspert et al. 2016). This still seems to apply for (+)RNA synthesis with the same or similar sequence context, but, in general, the proximal nucleotide context and polymerase complex also play a role and, dependent on context, insertional slippage can be detected at high frequency during (−)RNA synthesis at both types of HH (A6 or U6) (Figs. 3 and 4).

According to the current understanding of positive-strand RNA virus replication, the synthesis of (−)RNA occurs probably only once per VRC and involves melting of intramolecular secondary structure in the template (+)RNA ahead of the polymerase. However, (+)RNA strands are synthesized in multiple rounds, at least 100- to 1,000-fold higher abundance than template (−)RNA, and synthesis involves continuous strand displacement of the previous replication round (+)RNA strand in the (+)RNA:(−)RNA replication intermediate duplex. This fundamental difference between the replication complexes during (−)RNA and (+)RNA synthesis is the most probable cause of differences in the levels of TWI generation and, with caution, also the substitution levels between the (−)RNA of ST3 and (+)RNA of ST3U (Fig. 3E and F). The ST3 and ST3U are reverse complements of each other and so the polymerase encounters identical nucleotide sequences during ST3 (+)RNA and ST3U (−)RNA synthesis (and vice versa), but the outcome is clearly different. The nucleotide sequence of the slip site in this comparison is identical in regard of the detected RNA product. This demonstrates that strand displacement during (+)RNA synthesis can impact slippage and that the polymerase complex during (−)RNA synthesis is probably more relaxed, leading to a more promiscuous outcome. A highly hypothetical mechanism of removal of (−)TWI from further replication could be the detection of bulged nucleotides in the double-stranded RNA replication intermediate by some viral protein, followed by the inactivation of the particular VRC.

### Substitution mutations at flanking −1 and +7 or +13 positions support the slippage model

During cell-to-cell and systemic movement, plant viruses go through many cells and replication cycles, which generate genetic bottlenecks. While the detected TWI are most likely produced *in situ* at the site of replication or transcription (in systemically infected cells), the substitutions produced at the 2ndGA6 site via to-fro slippage are neutral to the virus and can originate from previous replication cycles and, theoretically, even reach 100%, e.g., if substitutions emerge early in infection and virus movement bottlenecks randomly impact native genomes. Therefore, the substitution data must be interpreted with caution. Tofro slippage during positive- or negative-strand synthesis causes a substitution at position +7 or −1, respectively (Olspert et al. 2016). The occurrence of to-fro slippage is also limited to certain nucleotide combinations (Olspert et al. 2016), for example, ST3 and ST3U (Fig. 1C). Mutants with an ‘A’ hexamer in the positive strand followed by ‘G’ in position +7 (i.e., TuMV_WT and ST3) have a propensity for +7 substitutions, whereas the corresponding reverse complement mutants (TuMV_RC and ST3U) produce −1 substitutions (Figs. 1C and 3B and F). This strong trend reversal further supports the proposed mechanism of to-fro slippage. Of all the tested slip-site sequences, only TuMV_RC showed a different distribution of substitution mutations between (+)RNA and (−)RNA populations (Figs. 3B and F and 4C and D). Since substitutions at 2ndGA6 are expected to be selectively neutral, seeing similar (+)RNA and (−)RNA substitution levels for most mutants makes sense. Unexpectedly, TuMV_RC showed enhanced insertion frequency and reduced −1 position substitution frequency in (−)RNA compared with (+)RNA. However, *de novo* generation of −1 substitutions via to-fro slippage can only occur during (+)RNA synthesis. Therefore, it is puzzling as to where the excess of −1 substitutions in the (+)RNA pool originate. Possibilities include positive selection for this substitution (which contradicts neutrality at the 2ndGA6 site) or a spurious mutational process at this site in the (+)RNAs that is unrelated to to-fro slippage. Although unlikely, we also cannot rule out a technical artifact specific to the TuMV_RC sequence.

Much to our surprise, the designed to-fro slippage–inhibiting mutation of the +13 (+7 equivalent) ‘G’ to ‘C’ in U6A6_noG did not perform as expected. In the U6A6_noG mutant, to-fro slippage at AAAAAAC (+ 13) would produce an A:G purine:purine mispairing in the nascent:template RNA duplex (instead of the A:C purine:pyrimidine mispairing in the U6A6 mutant), but this did not reduce the substitution level at that position (Fig. 4C), in contrast to what one would expect based on previous observations (Olspert et al. 2016). However, with A6U6_noG, the same + 13 position is preceded by the run of Us, so that to-fro slippage would lead to a U:G pyrimidine:purine mispairing (instead of a U:C pyrimidine:pyrimidine mispairing, as in A6U6), which is more favorable (Fig. 4D) (Olspert et al. 2016). In this case the expected pattern of reduced substitutions at position + 13 in A6U6 compared with A6U6_noG was observed (Fig. 4D).

### Environment conditions, infection stage, and host plant impact slippage

There are probably several factors responsible for the higher insertion level and infection tolerance at elevated temperature. For instance, the viral RNA polymerase may be more promiscuous and, therefore, also more slippage prone. However, it is likely that alterations in host gene expression contribute more to the general outcome. It is known that NMD is downregulated in response to stress (e.g., heat) in both mammals and plants (Shaul 2015). At the same time, many plant pathogen-defense response factors are endogenous NMD substrates and are suppressed by NMD under normal conditions (Rayson et al. 2012). Therefore, heat stress suppresses NMD, which might regulate the level of TWI, but elevated levels of pathogen response proteins are expressed due to NMD dampening, resulting in suppression of the virus. It is difficult to assess which contributes more to the change in TWI—changes in virus replication or changes in host response to infection. Together with the detected differences in viral RNA levels (Supplementary Fig. S2), the overall conclusion is that elevated temperature and prolonged infection modestly but significantly influence the ratio of TWI at the native site WT_GA6 independent of viral load. Seeing stable slippage levels with varying viral RNA loads suggests the absence of more complex regulation mechanisms of slippage frequency dependent on viral RNA or protein levels. At the 2ndGA6 site the sequence-related differences outweighed potential effects of temperature and infection stage.

We also noticed differences in slippage frequencies when other host species were used. Most of our experiments were in *Nicotiana benthamiana* (Figs. 1, 2, 3, and 4), but the NMD-related parts required the use of *Arabidopsis thaliana*, in which only TuMV_WT and ST viruses were tested (Fig. 5). The difference is most prominent with virus TuMV_WT, which contains the native *pipo* slip sequence at both the WT_GA6 and 2ndGA6 sites. In *N. benthamiana*, we see roughly similar TWI levels at both sites (Figs. 1B and C, 2A, B, and C, 3A and B), whereas in *A. thaliana*, we constantly see roughly twofold more at the 2ndGA6 site (Fig. 5). Also, for the ST mutant, the TWI level at the 2ndGA6 site peaks at a slightly lower value in *A. thaliana* than in *N. benthamiana* (about 4% vs. about 6%). We must, of course, take into account the potential impact of the different photoperiod used for *A. thaliana*, but, based on TuMV_WT, it is more likely that some host-specific difference is responsible for the elevated TWI level seen at the 2ndGA6 site.

### NMD as a potential regulator of TWI levels

Our data suggest that TWI from both sites and also native genomes associate with complexes containing UPF1 and are presumably subsequently degraded. However, TWI are more frequently detected with UPF1 only when TWI are produced at a high level, such as with the ST mutant. Therefore, we conclude that, in respect to the balance between synthesis and degradation of available TWI, NMD is a contributing negative regulator of the detected insertion level only when the slip site produces high numbers of insertions. This regulation, of course, applies only to the translated RNA subpopulation, as (−)RNA inside VRCs and (+)RNA inside virions are not available for NMD. Due to limited sample quantity, it was not feasible to purify polysomes from the same material that was used for IP, so the comparison is between total (+)RNA and UPF1-associated (+)RNA. Therefore, the actual amount of NMD-available “input” TWI can be masked by virion-protected TWI, if it is present in high amounts. However, our previous data suggest that the differences in TWI levels between polysome, virion, and total (+)RNA are not large (Fig. 3). The difference between polysome and virion TWI levels is biggest at the ST 2ndGA6 slip site, which might, in light of the other ST-related NMD data, be a sign of NMD reducing the amount of TWI being translated (Fig. 3D).

Potentially the native *pipo* stop codon may have evolved NMD-protection elements that have been described for other RNA viruses (Popp et al. 2020), which would stabilize WT_GA6 (+)RNA TWI. This type of protection would be absent from the artificial 2ndGA6 site, thus making (+)RNA TWI from that site susceptible to NMD and reduced TWI levels. However, our data from Co-IP and UPF1 knockdown infection experiments do not fully support this. For the TuMV native *pipo* slip sequence, we see about 1.5-to 2-fold more TWI associated with UPF1 than in input RNA at either site, WT_GA2 or 2ndGA6 (Fig. 5A and B). There is also no detectable difference in TuMV_WT TWI levels between the sites in control and NMD-compromised plants (Fig. 5C and D). If the native slip site has some NMD-evasion mechanism, its impact is not detectable. However, we notice the impact of NMD on 2ndGA6 site TWI and viral RNA levels in some ST samples (Fig. 5B to D; Supplementary Fig. S3), which suggests that TuMV RNAs are not fully protected from NMD (e.g., by elements embedded in the RNA or in *trans* disabling of NMD via virus-host interactions). Perhaps TuMV transcriptional slippage has co-evolved with NMD and the virus can cope with so little TWI for P3N-PIPO production that it does not need additional RNA embedded NMD-protection elements.

### Conclusion

In this paper, we have investigated the dynamics of TuMV transcriptional slippage during infection and in different environmental conditions. We have also begun the characterization of the biogenesis of TWI. The most intriguing remaining questions involve the differences in (+)TWI and (−)TWI levels and their origins, such as whether (+)TWI are produced solely *de novo* or arise also from replication of (−)TWI and whether there are (additional) mechanisms that exclude one or both (−)TWI and (+)TWI from further replication. These studies could be expanded, given potyviral replicon systems that can be manipulated to separate (+)RNA and (−)RNA synthesis, though, unfortunately, attempts to generate such systems have, so far, been unsuccessful.

## Materials and Methods

### Viruses, plasmids, and *A. thaliana* lines

*Turnip mosaic virus* expressing GFP, GenBank accession EF028235 (all TuMV numbering below corresponds to this accession), based on isolate UK1, in an agroinfiltration-competent plasmid with an extra transcriptional slippage cassette after *gfp*, described by Olspert et al. (2016), was used as the basis for new constructs. Novel second-GA6 slip sites (Figs. 1A and 4A) were introduced by oligo cloning between *Sma*I and *Nhe*I restriction enzyme sites, as described by Olspert et al. (2016). A GFP-free TuMV variant corresponding to TuMV UK1, GenBank accession AF169561, was generated from the GFP-expressing clone described above, using standard cloning methods.

*A. thaliana* tagged UPF1, UPF1-FLAG-HA, and UPF1-GFP lines were obtained from D. Garcia (Chicois et al. 2018). UPF1 T-DNA knockdown lines *upf1-5* (SALK_112922) and *upf1-3* (SALK_081178) were obtained from the Nottingham Arabidopsis Stock Centre.

### Inoculation, RNA purification, and Co-IP

*N. benthamiana* plants were grown under a 16-h photoperiod at 22 or 30°C (200 μE m^2^ s^−1^ of photosynthetically active radiation) and 60% humidity. *A. thaliana* was grown at 22°C under the same conditions, but 3 days prior to inoculation or agroinfiltration, it was transferred to a 12-h photoperiod, which was maintained for the remainder of the experiment. Three- to four-week-old plants were inoculated by agroinfiltration as described previously (Olspert et al 2016). For *A. thaliana* experiments that were sampled at 14 DPI, plants were mechanically inoculated, using Carborundum with TuMV-infected *N. benthamiana* sap, as in our experience mechanical inoculation results in earlier and faster TuMV infection progression in *A. thaliana*. Samples of systemically infected leaves were collected at the desired times. Plant tissue was frozen in liquid nitrogen and was homogenized, and total RNA was extracted as described by Oñate-Sánchez et al. (2008). Co-IP was carried out using μMACS GFP or HA isolation kits (MACS purification system, Miltenyi Biotech), according to manufacturer instructions as described by Chicois et al. (2018).

### Virus and polysome purification

Virions were purified from systemically infected leaves as by Baratova et al. (2001). Polysomes were purified as by Jackson et al. (1976), with modifications as described by Berry et al. (1988).

### Reverse transcription PCR (RT-PCR) and high-throughput sequencing

Next-generation sequencing (NGS) amplicon libraries were generated via RT-PCR, with adapter-containing primers as described by Olspert et al. (2016), with modifications. We used longer viral target sequences for NGS of the second-GA6 and WT-GA6 sites—TuMV-GFP nt 1,895 to 1,994 and 3,814 to 3,936, respectively. Negative strand–specific RT-PCR was performed with tagged primers, followed by Exonuclease I treatment to avoid primer carryover as described by Purcell et al. (2006), with modifications. In order to further reduce false priming, the RT reaction was not cooled on ice after denaturation at 70°C and primer annealing but was cooled to and kept at 55°C (which was also the RT incubation temperature) throughout the process. In validation experiments, these measures ensured strand-specific detection only with RevertAid reverse transcriptase (Thermo), which showed absence of product in reactions with excess of the other strand but without the desired target strand (data not shown). Due to low abundance of (−)RNA in comparison to (+)RNA, virion RNA, and polysome-associated RNA, the templates (RT reactions) were diluted for the latter three in order to keep PCR cycles constant between all tested RNA subpopulation samples. In addition to more amplification cycles, the preparation of (−)RNA libraries required an additional PCR step (PCR1 + primer degradation resulting in PCR2) for adapter inclusion. We have seen in our experiments that changing the number of amplification cycles and the number of separate PCR steps for adding the adapters (PCR vs. PCR1 + primer degradation resulting in PCR2) does not change the detected transcriptional slippage characteristics significantly (data not shown). However, for absolute bias-free comparison, all libraries ((−)RNA, (+)RNA, polysome RNA, and virion RNA) were prepared with the same number of cycles and PCR steps. After amplification, libraries were polyacrylamide gel electrophoresis–purified, were quantified fluorometrically with Qubit dsDNA HS kit (Life Technologies), were normalized, and were sequenced, using the NextSeq500 platform (Illumina). Reads were checked for quality, were clipped for adapter sequence, and were demultiplexed based on site and library (second-GA6 and WT-GA6), using the FASTX Toolkit (Hannon lab). Reads containing Ns, truncated reads, obvious contaminating reads from other libraries (errors in indexing), and less abundant reads (count less than 3, well below 0.01% of total reads) were not included in the analysis. Reads were subsequently analyzed for insertions, deletions, and substitutions using custom scripts, mostly utilizing BioPython (Cock et al. 2009).

## Supplementary Material

Supplementary Material
