# Supplementary material for: Turnip Mosaic Virus Transcriptional Slippage Dynamics and Distribution in RNA Subpopulations
Source: Mol Plant Microbe Interact. Author manuscript; Available in PMC 2026 Mar 10. (PMC7618844; doi:10.1094/MPMI-03-22-0060-R)
Supplement: Supplementary Material [file EMS212720-supplement-Supplementary_Material.pdf]

Supplementary figure S1

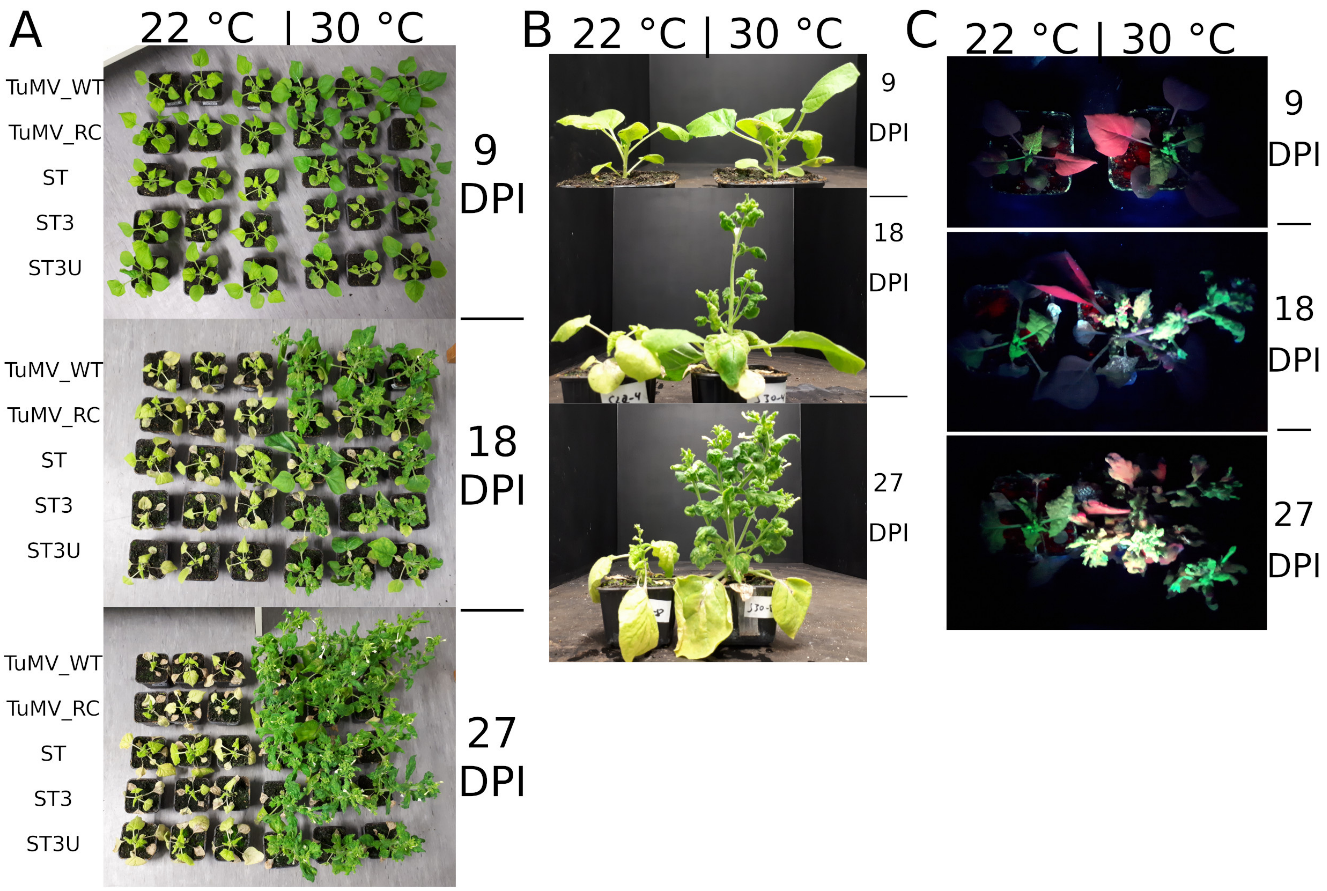

Supplementary fig. S1. Overview of infection symptoms at different temperatures. *Nicotiana benthamiana* plants were inoculated with TuMV constructs and grown at 22 °C or 30 °C. (**A**) Top view of plants infected with various virus mutants in rows (name on left), at both temperatures (shown above), three plants for each temperature. The time point for the image is indicated on the right. (**B**) Example side views of plants infected with virus ST at either temperatures, for each time point. (**C**) Example top view under UV light of plants infected with virus ST at either temperatures, for each time point.

Supplementary figure S2

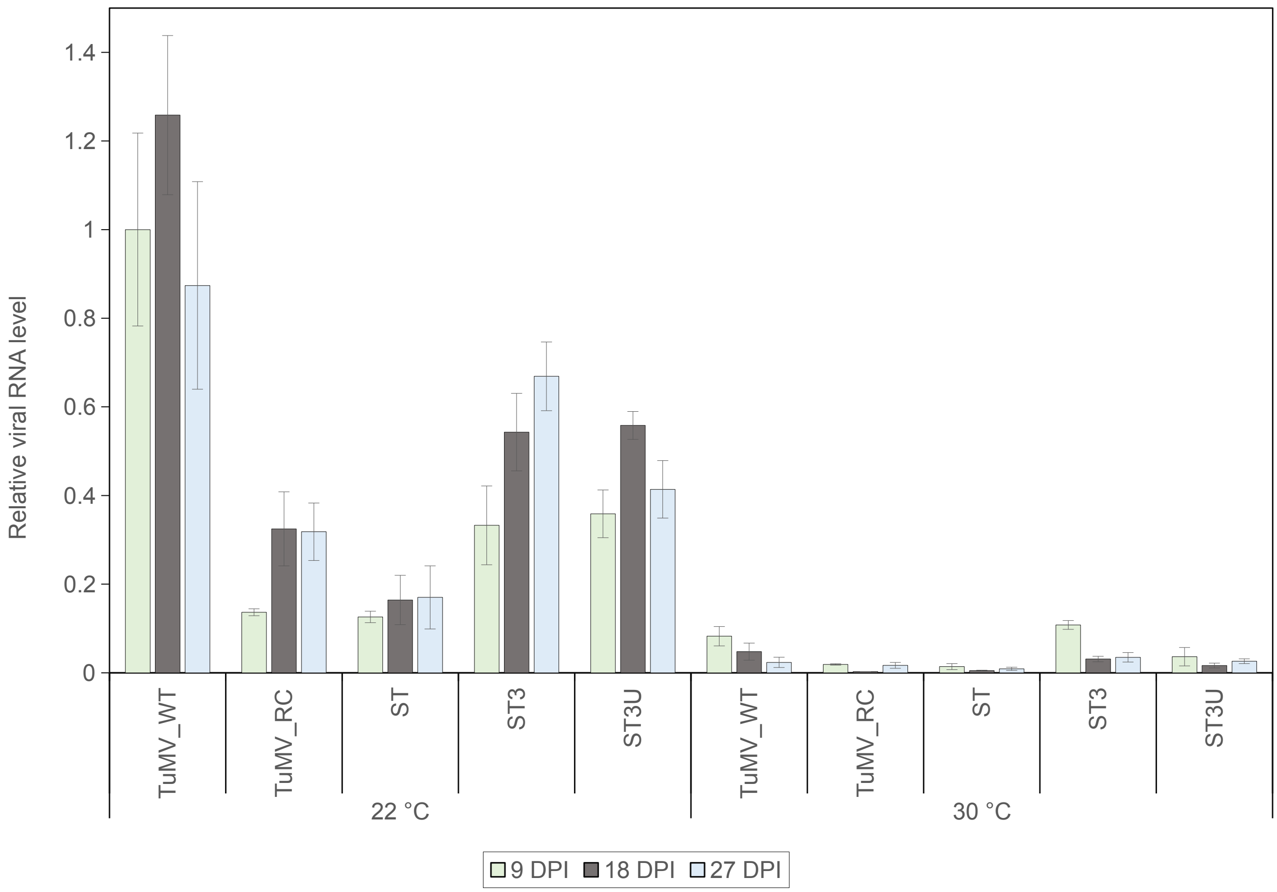

Supplementary fig. S2. Relative levels of viral RNA in systemically infected top leaves and flower buds at 9, 18 and 27 days post inoculation (DPI) for plants grown at 22 °C or 30 °C. Total RNA was extracted and subjected to RT-qPCR. Viral RNA levels were normalized using 18S. Data were log-transformed and mean-centered. The standard error of the mean is shown with whiskers.

Supplementary figure S3

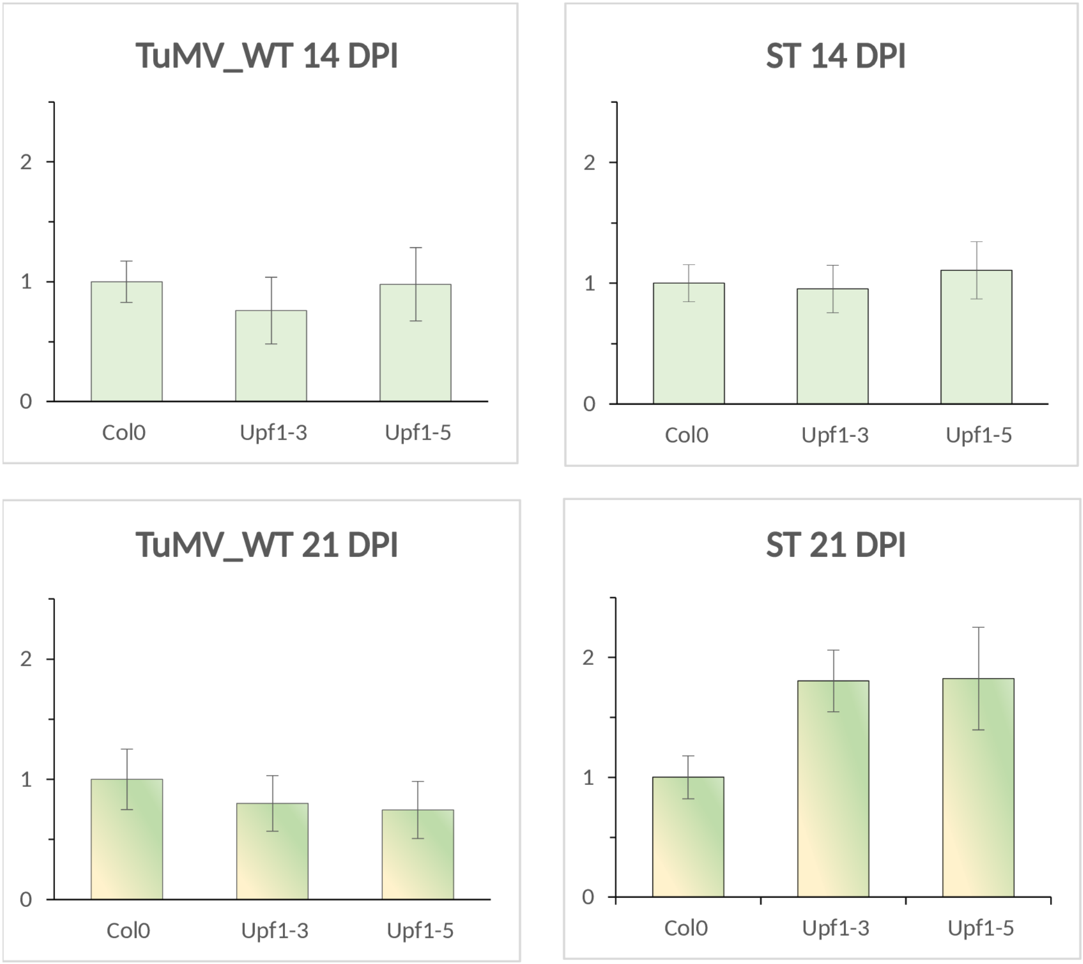

Supplementary fig. S3. Relative level of viral RNA in systemically infected top leaves and flower buds at 14 and 21 DPI for *Arabidopsis thaliana* Col0 and UPF1 T-DNA knockdown lines Upf1-3 and Upf1-5. Total RNA was extracted and subjected to RT-qPCR. Data were log-transformed, mean-centered and autoscaled. The standard error of the mean is shown with whiskers.

Table S1. Frequencies of the substitutions of position -1 and +7 to the nucleotide identical to the proximal homopolymeric hexamer in sense orientation for all experiments presented in the study.

Site	2ndGA6 mutant	Proportion of substitutions at position -1 to the nucleotide of the homopolymeric hexamer (A or U)				Proportion of substitutions at position +7 (or equivalent) to the nucleotide of the homopolymeric hexamer (A or U)			
		Mean, %	Median, %	Standard deviation, %	Number of samples	Mean, %	Median, %	Standard deviation, %	Number of samples
WT_GA6		69.9	77.8	23.4	378	56.6	60.6	24.7	378
2ndGA6	TuMV_WT	80.2	94.7	21.3	84	64.9	64.9	13.2	84
2ndGA6	TuMV_RC	89.7	89.1	6.7	37	16.8	11.7	20.5	37
2ndGA6	ST	81.2	88.9	18.6	95	67.4	70.6	24.1	95
2ndGA6	ST3	65.9	73.6	22.9	37	94.7	94.7	3.1	37
2ndGA6	ST3U	97.5	98.0	2.0	37	90.5	98.2	23.0	37
2ndGA6	U6A6	98.5	98.9	0.8	10	40.3	45.1	19.5	10
2ndGA6	U6A6_noG	98.3	98.7	1.0	10	84.8	84.7	1.5	10
2ndGA6	A6U6	80.2	83.3	11.5	10	84.0	84.9	7.9	10
2ndGA6	A6U6_noG	82.8	85.3	11.1	10	7.2	5.7	4.4	10

Table S2. Primer and probe sequences used for RT-qPCR

Primer/probe	Sequence
TAG3-2108-R	5′-GCAAGCAGAAGACGGCATACGACAGTGACATCTAGCCCTGAATAG-3′
TAG3	5′-GCAAGCAGAAGACGGCATACGA-3′
Fl-1895-F	5′-AATAAATCATAAGTAACAGCTGCTGGGATTACAC-3′
TuMV-P1 probe	5′-Fam-TCCAGAAGTTGGCTCCCGCTGCACCT-BHQ-1-3′
EF1α-F	5′-TCCCAGGTCATCATCATGAACCA-3′
EF1α-R	5′-CAACAACCATGGGCTTGGT-3′
EF1α probe	5′-Tet-CCTTACCAGAACGCCTGTCAATCTTGG-BHQ-1-3′
SAND_At-F	5′-CCATATGCGTTAAGGCAAGCT-3′
SAND_Nb-F	5′-GCTTATGCGACAAGGCAAGCT-3′
SAND-R	5′-GGTGAGAAAGATTCTGATGTCCT-3′
SAND probe	5′-JOE-TCAGTCTTGCTGGTGCACAGAAAGC-BHQ-1-3′
18S-F	5′-GTAGTCCATGCCGTAAACGATG-3′
18S-R	5′-TGTTCATATGTCAAGGGCTGG-3′
18S probe	5′-JOE-TGCGACCGTACTCCCCAGGCGGA-BHQ-1-3′

Supplementary methods

RT-qPCR. Equal amounts of DNase I-treated RNA, which were checked for residual DNA contamination by PCR, were used for cDNA synthesis with RevertAid Reverse Transcriptase (Thermo Fisher Scientific) and a mixture of gene-specific primers (Table S2). qPCR reactions were performed with suitable primers and TaqMan probes in 7 µl in duplicates with HOT FIREPol Probe qPCR Mix Plus (ROX) (Solis Biodyne) using a 7900HT Fast Real-Time PCR System (Applied Biosystems).

Three reference genes were used for normalization where possible: EF1α, SAND and 18S. Data were log-transformed, mean-centered and autoscaled as suggested by Willems et al (2018). Data were back transformed for graphical representation.

Supplementary references

Willems,E., Leyns,L., Vandesompele,J. (2008) Standardization of real-time PCR gene expression data from independent biological replicates. Anal Biochem 379:127–129.
